# ﻿*Thismialatiffiana* (Thismiaceae), an unusual new species from Terengganu, Peninsular Malaysia

**DOI:** 10.3897/phytokeys.188.77061

**Published:** 2022-01-18

**Authors:** Mat Yunoh Siti-Munirah, Nikong Dome

**Affiliations:** 1 Forest Research Institute Malaysia, 52109 Kepong, Selangor, Malaysia Forest Research Institute Malaysia Kepong Malaysia; 2 DigitalDome Photography, 21500 Permaisuri, Terengganu, Malaysia DigitalDome Photography Terengganu Malaysia

**Keywords:** Hulu Nerus Forest Reserve, lowland dipterocarp forest, mycoheterotrophic, taxonomy

## Abstract

*Thismialatiffiana* Siti-Munirah & Dome, a new species from Terengganu, Peninsular Malaysia, is described and illustrated. The new species differs from all other species of *Thismia*, described so far, in having golden trichomes that are present on the outer surface of its floral tube and mitre, as well as pyramidal protuberances on the inner surface of the floral tube. Additionally, it is remarkable in its supraconnective apically bearing two long trichomes. *Thismialatiffiana* is assigned a preliminary conservation status as Critically Endangered (CR) according to IUCN Criteria.

## ﻿Introduction

*Thismia*Griffith (1844) is a genus of small mycoheterotrophic herbs found on the forest floor that usually go unnoticed due to their size and habit. *Thismia* belongs to the family Thismiaceae, which consists of 95 accepted species, of which 16 species have been recognised in Peninsular Malaysia. In particular, the State of Terengganu currently has eight reported species of *Thismia*: *T.alba* Holttum ex Jonker, *T.arachnites* Ridl., *T.aseroe* Becc. (all three Siti-Munirah and Dome 2021), *T.domei* Siti-Munirah (Siti-Munirah and Dome 2019), *T.javanica* J.J.Sm. (Siti-Munirah and Dome 2019), *T.ornata* Dančák, Hroneš & Sochor (Siti-Munirah and Dome 2021), *T.sitimeriamiae* Siti-Munirah, Dome & Thorogood (Siti-Munirah et al. 2021) and *T.terengganuensis* Siti-Munirah (Siti-Munirah and Dome 2019).

In December 2019, the second author discovered an odd-looking *Thismia* at Hulu Nerus Forest Reserve (FR) located in Setiu District, State of Terengganu, eastern Peninsular Malaysia. The specimens have been deposited to the Kepong Herbarium (KEP). After comparing it with all the species of *Thismia* known to date, we concluded that this plant does not match any of them. Herein, we describe it as a new species, *Thismialatiffiana* Siti-Munirah & Dome.

## ﻿Materials and methods

**Map 1.**
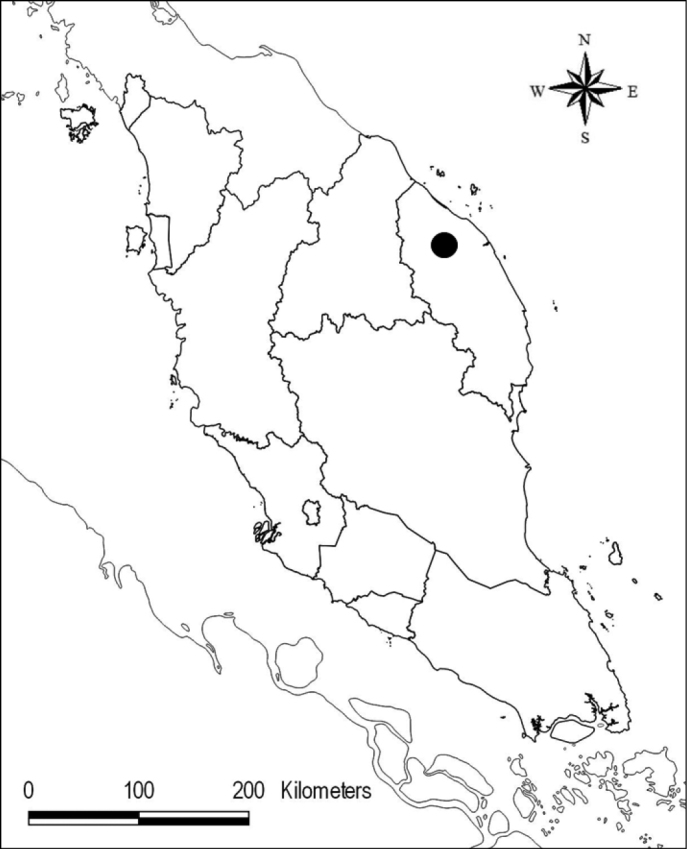
Map of Malay Peninsula with Hulu Nerus Forest Reserve (●), the type locality of *Thismialatiffiana*.

This study is based on material collected in December 2019 in Hulu Nerus FR, Setiu District, Terengganu (Map 1). Morphological characteristics were studied using a stereomicroscope and high-resolution macrophotography. Measurements were taken from both fresh and liquid-preserved materials. The specimen details were compared with original drawings and descriptions in the protologues of *Thismia* species around the world.

## ﻿Taxonomic account

### ﻿ Thismia latiffiana

Taxon classificationPlantaeDioscorealesBurmanniaceae

Siti-Munirah & Dome sp. nov.

2D23FAB4-1B4E-52C8-AC43-E98396CF8278

urn:lsid:ipni.org:names:77248767-1

Figures 1 , 2 , 3

#### Diagnosis.

*Thismialatiffiana* differs from all its congeners by the following combination of traits: the presence of golden trichomes on the outer surface of floral tube and mitre, outer tepals absent, inner tepals form a mitre without appendages, an inner surface of floral tube covered by pyramidal protuberances and supraconnective bilobed with each lobe terminated by a long, needle-like trichome.

**Figure 1.**
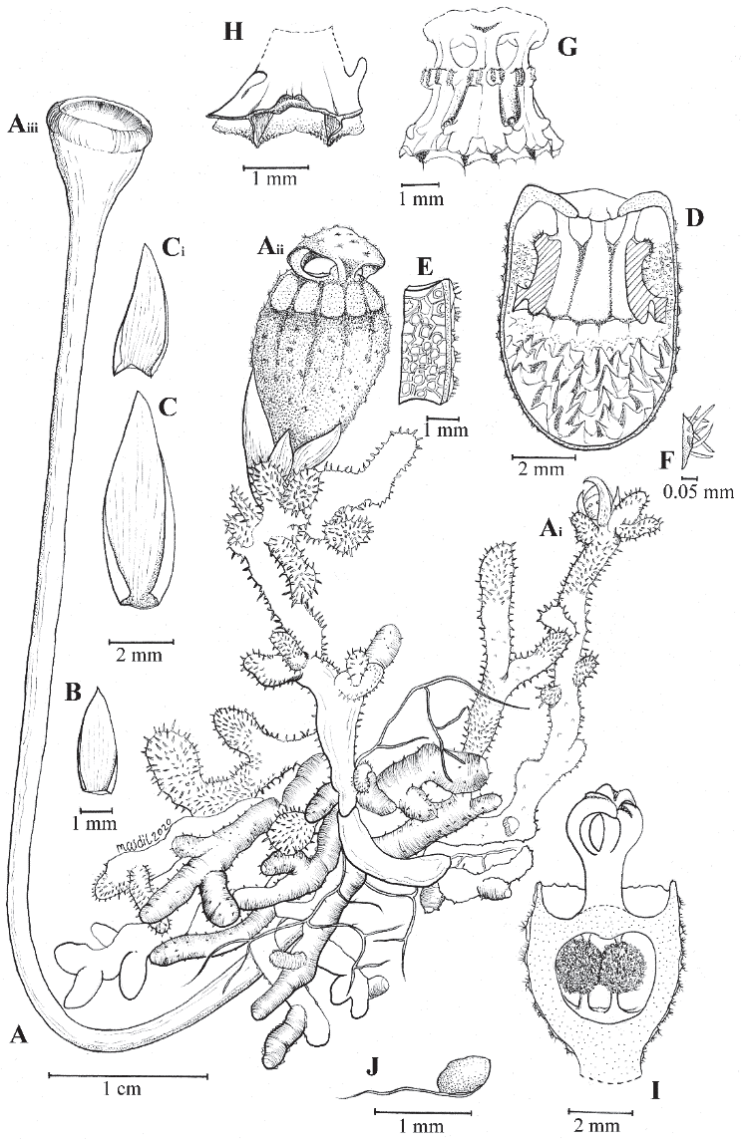
Illustration of *Thismialatiffiana***A** habit, showing roots, young bud (Ai; note stems covered with trichomes), mature flower (Aii) and fruit (Aiii; note glabrescent stem with trichomes detached) **B** Leaf (adaxial) **C** bract (adaxial), smaller bract (Ci) **D** longitudinally dissected floral tube showing inner (abaxial) view of stamens and apical parts of connectives **E** portion of inner surface of floral tube (upper part) **F** trichomes on outer surface of floral tube; **G** outer (adaxial) view of stamens showing lateral appendages **H** stamen, view from below **I** gynoecium, longitudinal section, showing pistil with trilobed stigma and ovary **J** seed. All from *FRI94686* (spirit material). Drawings by Mohamad Aidil Noordin.

#### Type.

Malaysia. Peninsular Malaysia: Terengganu: Setiu District, Hulu Nerus Forest Reserve, approximately 220 m elev., 4 February 2020, *Siti-Munirah & Dome Nikong FRI94686* (holotype KEP!, spirit collection, barcode no. 280004).

**Figure 2.**
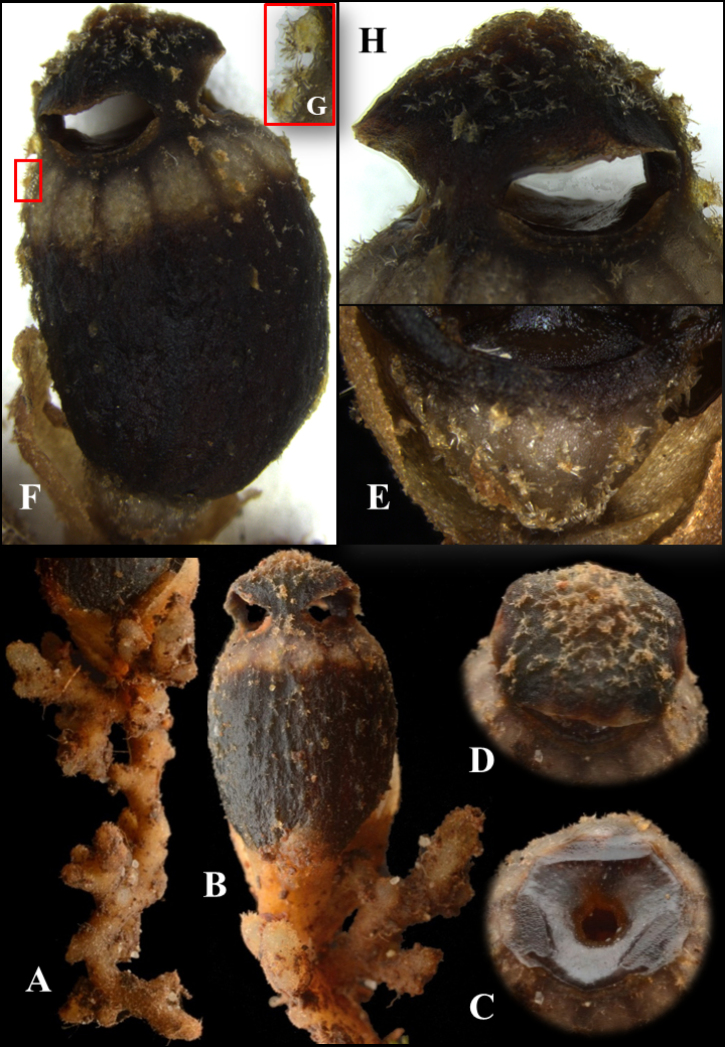
*Thismialatiffiana*, external morphology **A** roots **B** mature flower **C** apical part of floral tube with opening aperture **D** apex of mitre **E** ovary, side view **F** mature flower **G** trichomes resembling spines on an areole of cacti **H** mitre, side view. All from *FRI91117*: **A, B, C, D***FRI94686*: **E, F, G, H**. Photos by Dome Nikong (**A–D**) and Siti-Munirah MY (**E–H**). Images not to scale (see dimensions in description and Figure 1).

***Herb*** achlorophyllous, approximately 12 mm tall. ***Roots*** loosely coralliform, compressed, light brown, surface covered with trichomes. ***Stem*** very short or absent, obscured by trichomes during flowering. ***Leaves*** spirally arranged, crowded, scale-like, triangular, apex acute to acuminate, margin entire, 2–8 mm long and 1.5–2 mm wide, brown. ***Bract*** similar to leaves. ***Flowers*** actinomorphic, solitary; floral tube 1.2 cm long, ellipsoid to ovoid, widest in middle part (7–7.3 mm in diameter), in upper part ca. 6.4 mm wide and at base ca. 6 mm wide, black or dark brown, whitish in upper part with round to oblong sectors (opposite each anther thecae), separated by blackish-brown stripes; surface partially covered with individual unbranched trichomes, stellate trichomes or unbranched trichomes crowded on warts (resembling spines on areoles of cacti); inner surface covered with very thick pyramidal protuberances arranged longitudinally in each section continuously, brown to black at middle towards base, upper part reticulate, light brown. ***Annulus*** at apical part of floral tube, dark brown, broadly funnel-shaped, ca. 2.7 mm wide, margin 6-lobed, glabrous, smooth, and thick. ***Outer tepals*** absent. ***Inner tepals*** 3, dark brown to blackish, erect and turning inwards, connate at the top forming a mitre without any appendages; ***mitre*** black or dark brown, on outer surface partially covered in a patchy manner with individual unbranched trichomes, stellate trichomes or unbranched trichomes crowded on warts (resembling spines on areoles of cacti); glabrous, smooth, blackish-brown on inner surface; mitre openings 3, ca. 3.5 mm × 5.2 mm. ***Stamens*** 6, pendent from the apical margin of the floral tube, dark brown; filaments free, short; connectives broad, flattened, laterally fused together and skirt-like, trigonous, ca. 2.4–2.7 mm × 1–1.4 mm, somewhat raised below thecae, flat on the side pointing to the centre of flower (towards apex); supraconnective apex 2-lobed, each lobe with solitary transparent needle-like trichomes ca. 0.5–0.6 mm long; ***thecae*** yellowish, surrounded by tufts of hairs at middle part; ***lateral appendage*** protruding towards floral tube, quadrangular, equalling the apex of supraconnective, with a horn-like projection arising from each side, margin shallowly dentate and sparsely hairy; ***interstaminal glands*** inserted on the lines of fusion between connectives. ***Ovary*** inferior, bowl-shaped, whitish-brown, warty, enveloped by bracts and leaves. ***Style*** ca. 0.8–0.9 mm long × 0.5–0.8 mm wide; ***stigma*** deeply trilobed; lobes curved inwards, ca. 2.2 mm long, surface papillose, dark brown. ***Fruit*** a cup- or bowl-shaped or pyxidium capsule, 2.6 mm in diameter, dark brown to blackish-brown, borne on an elongated pedicel up to 7–10 cm long, distally covered with old dusty trichomes, proximally glabrous.

**Figure 3.**
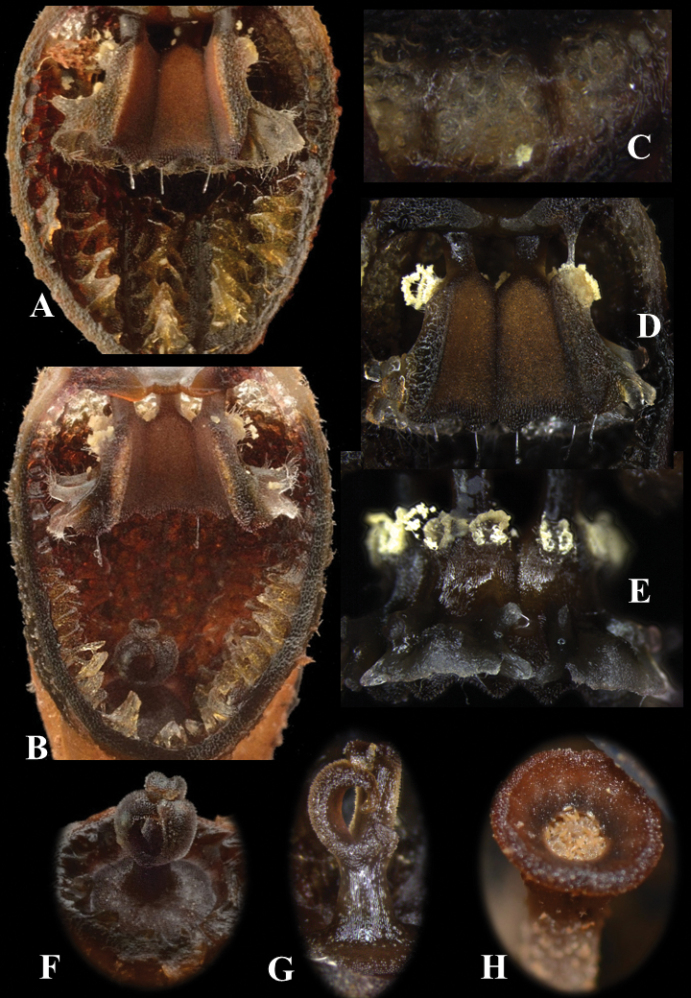
*Thismialatiffiana*: inner floral structure **A, B** floral tube, longitudinal section **B** shows pistil and inner surface of floral tube covered with pyramidal protuberances arranged longitudinally **C** floral tube, upper portion of longitudinal section **D** inner (abaxial) view of stamens showing supraconnectives **E** outer (adaxial) view of stamens showing lateral appendages **F** pistil, top view, showing stigma **G** pistil, side view **H** fruit, top view. All from *FRI91117*: **A, B, F***FRI94686*: **C, D, E, G, H**. Photos by Dome Nikong (**A, B, F**) and Siti-Munirah MY (**C, D, E, G, H**). Images not to scale (see dimensions in description and Figure 1).

#### Additional specimen examined (paratype).

Peninsular Malaysia. Terengganu: Setiu, Hulu Nerus Forest Reserve, ca. 220 m elev., 26 December 2019, *Dome Nikong FRI91117* (KEP, spirit collection, No. barcode 280003).

#### Distribution.

Endemic to Peninsular Malaysia, Terengganu. Currently known only from the type locality.

#### Ecology.

**Figure 4.**
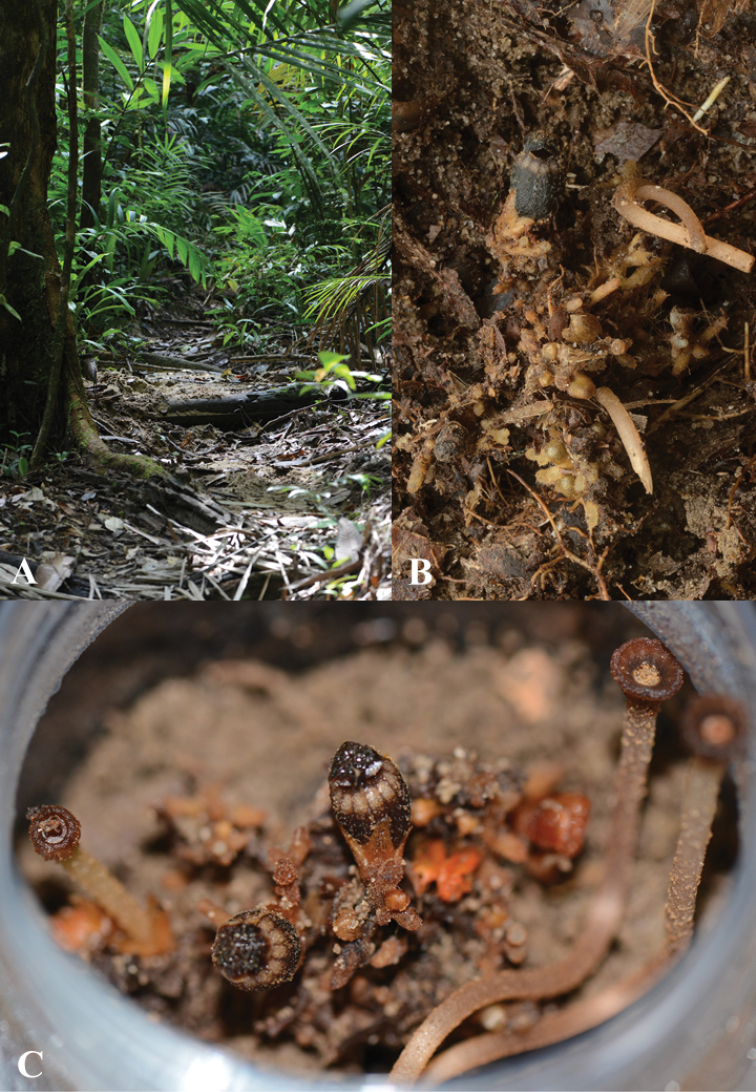
*Thismialatiffiana* Siti-Munirah & Dome **A** walking trail near the habitat **B** plant in its habitat **C** plant ex-situ. Photos by Siti-Munirah MY (**A, C**) and Dome Nikong (**B**).

In a lowland dipterocarp forest, on moist soil, under shade, near an open place (walking trail) (Figure 4) at elevation of 220 m. Flowering from December to February.

#### Etymology.

*Thismialatiffiana* is named in honour of Emeritus Professor Dato’ Dr. Abdul Latiff Mohamad, a prominent botanist and conservationist in Malaysia.

#### Vernacular name.

We suggest a local name as ‘Thismia burung hantu’ in Malay, due to its appearance resembling an owl (‘burung hantu’ = owl).

#### Preliminary conservation status.

Critically Endangered (CR B2ab(ii,iii)). Following the IUCN Red List Categories and Criteria (IUCN 2019), this species is assessed as Critically Endangered because it is currently known only from a single locality, where only two individuals were observed. The locality is within the Forest Reserve, but it is exposed to tourism activities within the Lata Payung Recreational Forest and Gunung Sarut. The habitat of the species is near the main trail from the entrance of Lata Payung to the ‘blue pool’ towards Gunung Sarut. Efforts to trace this species in the surrounding area were unsuccessful. Considering its small population and the threats to its microhabitat, *T.latiffiana* is assessed as Critically Endangered.

#### Notes.

As follows from its morphology, *T.latiffiana* belongs to the sectionSarcosiphon (Blume) Jonker (Jonker 1938). In addition, following the identification key in Kumar et al. (2017), *T.latiffiana* is falling within the subgen. Thismiasect.Sarcosiphon due to the arrangement of inner tepals into a mitre, the absence of outer tepals and a mitre lacking a filiform appendage. Additionally, based on the phylogeny in Shepeleva et al. (2020), *T.latiffiana* should belong to or near to clade 3 for its coralliform roots, inner tepals fused into a mitre, absence of mitre foveae and absence of outer tepals.

In the sectionSarcosiphon, the gross morphology of *T.latiffiana* is similar to that of several other species, such as *T.brunneomitroides* Suetsugu & Tsukaya (Suetsugu et al. 2017), *T.brunneomitra* Hroneš, Kobrlová & Dančák (Hroneš et al. 2015), *T.crocea* (Becc.) J.J.Sm. (Beccari 1878) and *T.cladestina* (Blume) Miq. (Chantanaorrapint et al. 2015). These species share with *T.latiffiana* a brown flower colour and an erect mitre with three lateral holes. However, all these abovementioned species have a long stem during flowering and densely clustered coralliform roots and *T.latiffiana* differs from them in having a very short (almost lacking) stem during flowering and rather loose coralliform roots. The new species is unique amongst the known *Sarcosiphon* species in having several unparalleled traits. *Thismialatiffiana* is recognisably different from all its congeners by the presence of golden trichomes on the outer surface of the floral tube, the pyramidal protuberances on the inner side of the floral tube and the supraconnective terminating into two long trichomes.

In Peninsular Malaysia, the most similar species is *T.sitimeriamiae* as it also has a very short stem during flowering and the presence of simple or occasionally apically stellate trichome structure on the outer side of the floral tube. However, it has minute, but distinctly developed, outer tepals and inner tepals forming a flattish, umbrella-like mitre. Therefore, the overall morphology of both species is completely different. For the record, *T.latiffiana* have been discovered in the same Forest Reserve as *T.sitimeriamiae*. Further investigation should be carried to better understand their relationship.

Interestingly, another species of sect.Sarcosiphon has been reported from Peninsular Malaysia (Perak, Gunung Hijau) by Ridley who described it as Bagnisiacroceavar.brunnea Ridl. (Ridley 1907). Jonker (1938, 1948) pointed out that it is highly unlikely that the specimen from Perak can be attributed to *T.crocea* from New Guinea although he did not see any specimen. A possible holotype of Bagnisiacroceavar.brunnea (Ridley s.n., SING 0052732!) contains an illustration and single dried plant that has coralliform roots and almost lacks stem during flowering and it is, in general appearance, similar to *T.latiffiana*. Therefore, it is possible that both taxa are conspecific. However, the illustration differs from *T.latiffiana* by small processes on the top of the mitre. Additional fieldwork and research is needed to resolve whether these two taxa are conspecific or not.

## Supplementary Material

XML Treatment for Thismia latiffiana
